# Epigenetic Therapy Augments Classic Chemotherapy in Suppressing the Growth of 3D High-Grade Serous Ovarian Cancer Spheroids over an Extended Period of Time

**DOI:** 10.3390/biom11111711

**Published:** 2021-11-17

**Authors:** Michelle Bilbao, Chelsea Katz, Stephanie L. Kass, Devon Smith, Krystal Hunter, David Warshal, James K. Aikins, Olga Ostrovsky

**Affiliations:** 1 Virtua Gynecologic Oncology, Virtua Health, Voorhees, NJ 08043, USA; mbilbao@virtua.org; 2 Department of Obstetrics and Gynecology, Cooper University Health Care, Camden, NJ 08103, USA; katz-chelsea@cooperhealth.edu (C.K.); kass-stephanie@cooperhealth.edu (S.L.K.); 3 Department of Obstetrics and Gynecology, Division of Urogynecology, Cooper University Health Care, Camden, NJ 08103, USA; smith-devon@cooperhealth.edu; 4 Department of Statistics, Cooper Research Institute, Cooper University Health Care, Camden, NJ 08103, USA; hunter-krystal@cooperhealth.edu; 5 Department of Gynecologic Oncology, MD Anderson Cancer Center at Cooper, Cooper University Health Care, Camden, NJ 08103, USA; warshal-david@cooperhealth.edu; 6 Rutgers Cancer Institute of New Jersey, New Brunswick, NJ 08901, USA; ja1220@cinj.rutgers.edu; 7 Department of Surgery, Division of Surgical Research, Cooper University Health Care, Camden, NJ 08103, USA

**Keywords:** high-grade serous ovarian cancer, recurrent ovarian cancer, chemoresistance, platinum resistance, taxane resistance, spheroids, organoids, classic chemotherapy, epigenetic therapy, panobinostat

## Abstract

Recurrent high-grade serous ovarian cancer (HGSC) is clinically very challenging and prematurely shortens patients’ lives. Recurrent ovarian cancer is characterized by high tumor heterogeneity; therefore, it is susceptible to epigenetic therapy in classic 2D tissue culture and rodent models. Unfortunately, this success has not translated well into clinical trials. Utilizing a 3D spheroid model over a period of weeks, we were able to compare the efficacy of classic chemotherapy and epigenetic therapy on recurrent ovarian cancer cells. Unexpectedly, in our model, a single dose of paclitaxel alone caused the exponential growth of recurrent high-grade serous epithelial ovarian cancer over a period of weeks. In contrast, this effect is not only opposite under treatment with panobinostat, but panobinostat reverses the repopulation of cancer cells following paclitaxel treatment. In our model, we also demonstrate differences in the drug-treatment sensitivity of classic chemotherapy and epigenetic therapy. Moreover, 3D-derived ovarian cancer cells demonstrate induced proliferation, migration, invasion, cancer colony formation and chemoresistance properties after just a single exposure to classic chemotherapy. To the best of our knowledge, this is the first evidence demonstrating a critical contrast between short and prolonged post-treatment outcomes following classic chemotherapy and epigenetic therapy in recurrent high-grade serous ovarian cancer in 3D culture.

## 1. Introduction

Ovarian cancer comprises 3.4% of all female cancers worldwide, but is responsible for 4.4% of all female deaths due to cancer [1]. High-grade serous ovarian, fallopian tube, and primary peritoneal cancer are thought to originate from similar fallopian tube precursors; therefore, the term “HGSC” will serve to encompass all three of these tumors [2]. Currently, 50–75% of patients enter remission after primary surgery and chemotherapy for HGSC, but unfortunately, 65% of these patients recur [3]. HGSC deaths are largely due to widely metastatic and chemoresistant disease and, despite recent advances, nearly all patients who recur will succumb to their disease [4].

The first divide in the treatment of HGSC occurs in those that are cured upfront as opposed to those that recur. The next divide in the recurrent cohort is in those that respond to platinum agents and those that do not. Roughly 75% of patients with recurrence will recur at over 6 months following initial chemotherapy and are deemed to be platinum-sensitive [5]. The 10% that progress on chemotherapy are noted to be platinum-refractory, and the 15% that progress from 0 to 6 months are platinum-resistant [5]. Platinum-resistant and platinum-refractory tumors have the poorest prognosis, with a 10% response to single-agent chemotherapy [6]. Additionally, it can be very difficult to adequately debulk these patients because they often develop small micrometastases throughout the abdomen and pelvis [7]. Progression-free survival for these patients is 3–4 months, whereas median overall survival is 9–12 months [6]. Furthermore, as a result of repeated exposure, HGSC patients develop not only platinum resistance, but taxane resistance as well [8]. Therefore, we critically need to develop and utilize the most clinically relevant models which mimic the progression of disease in patients, and define newer, more effective therapeutic strategies that may help to save patients from recurrent HGSC.

The majority of preclinical studies in HGSC are based on a two-dimensional (2D) in vitro cell culture model that is easily constructed and interpreted in the laboratory setting [9,10]. Traditionally, 2D monolayer cultures are followed by animal models and then clinical translational studies. Unfortunately, many therapeutic agents, which are highly effective in 2D models, translate to up to a 95% failure rate in clinical trials [9,10]. One reason for this discrepancy is that HGSC does not grow in monolayer in vivo; it grows in 3D spheroids [11]. Furthermore, cells in 2D monolayers have very different properties from cells in 3D spheroid culture. Three-dimensional cells have different cell–cell communication and cellular matrix components that cannot be replicated in 2D culture [12]. Furthermore, cells in a 3D configuration secrete more growth factors than cells in monolayer [10]. In fact, some epithelial ovarian cancer cell lines, such as Caov-3 and Ovcar-3, proliferate more slowly and are more chemoresistant in 3D than 2D culture [10]. Furthermore, 3D structures demonstrate tumor features that cannot be replicated in 2D, for example, hypoxic and necrotic areas [13]. More importantly, studying HGSC 3D models is more relevant because they better mimic chemoresistance [10]. HGSC spheroids better maintain the tumor heterogeneity found in chemoresistant cell lines [14], whereas in vitro 2D culture can take 3 to 18 months to develop a chemoresistant cell lines [15]. Moreover, in 3D culture, chemoresistance develops much faster and more closely replicates the histological, biological and molecular frameworks found in patient-derived ovarian cancer tissue [10]. These 3D spheroids have been shown to be responsible for the micrometastases that are so difficult to eradicate in platinum-sensitive and platinum-resistant HGSC [7]. Furthermore, chemoresistant cancer stem cells are far more abundant in 3D cultures when compared to 2D cultures [16].

Therefore, 3D culture can be a challenge, but when it is mastered, the results can be reliably and accurately measured [17] and more closely mimic the pathophysiology found in patients than 2D culture because 3D cultures have a similar density to natural human tissues and also demonstrate a similar response to what is seen in solid tumors [11,18]. The morphology and cellular matrix of 2D culture is problematic for adequately studying the tumor microenvironment of HGSC, but so is the timing of experimentation. Chemotherapy for HGSC is typically given in 21-day cycles, whereas 2D experimentation is performed over a period of days. Therefore, extended kinetics are required to adequately study this chemoresistance problem. The typical 48- or 72-h kinetic in 2D monolayers is again convenient, but not clinically relevant [15]. The platinum and taxane chemotheraputics given in these 21-day cycles are protein-bound and then the remainder is excreted in 24–52 h [19]. The protein-bound portion has been found to remain in the body for several days in some studies and several months in others [15,20]. The effect of this low-level exposure on HGSC is largely unknown.

As previously stated, recurrent disease can be prolonged with chemotherapy, but is never curative. Epigenetic changes such as aberrant DNA methylation and histone acetylation are present in both intrinsic and acquired drug resistance in HGSC [21]. Specifically, both the hypo- and hyper-methylation of CpG islands have been associated with chemoresistance, cell cycle control, and apoptosis [21]. For example, when the promotors of oncogenes BRCA1 and BRCA2 are hypermethylated, they are silenced [22]. This inactivation of DNA repair fuels malignancies such as breast and ovarian cancer [23]. Similarly, the demethylation of tumor suppressor genes such as p53, MLH1, p16 and others also contributes to the genetic instability responsible for the development of HGSC [24]. These epigenetic changes provide the framework for the development of cancer chemoresistance, proliferation and metastasis [25]. Furthermore, there is evidence that epigenetic drugs, which modify these epigenetic changes, are not only directly cytotoxic, but can make classic chemotherapy more effective by reversing chemoresistance [26].

In our work, we explore the efficacy of classic chemotherapy and epigenetic therapies in both platinum-sensitive and platinum-resistant recurrent human ovarian cancer cells in a 3D culture model and compare it to the classic 2D monolayer culture. In constructing our experiments evaluating chemoresistance and recurrent ovarian cancer, we not only used a 3D model, but also evaluated these spheroids under different therapeutic strategies over a longer kinetic. Instead of looking at our spheroids over 24–48 h, we evaluated them over 7–14 days. Through investigating the prolonged post-treatment effect of our therapeutics on cancer growth, we uncover a behavior of HGSC that may more closely replicate the regrowth of cancer following chemotherapy in patients and is therefore more clinically relevant. Finally, we demonstrate that the significant changes in growth, migration and chemoresistance in 3D-derived HGSC following our prolonged kinetic with classic chemotherapy may be reversible with epigenetic intervention.

## 2. Materials and Methods

### 2.1. Therapeutic Agents

Paclitaxel, cisplatin, panobinostat (pano) and suberoylanilide hydroxamic acid (SAHA) were supplied in powdered form and stored at −20 °C (Table 1).

Paclitaxel and SAHA were supplied from Sigma-Aldrich (St. Louis, MO, USA). Cisplatin and panobinostat were supplied from Cayman Chemical (Ann Arbor, MI, USA). Stock solutions of paclitaxel, panobinostat, and SAHA were prepared using 100% dimenthyl sulfoxide (DMSO; Sigma-Aldrich). Aliquots were stored at −20 °C. Cisplatin was prepared using sterile normal saline and stored at −4 °C. Cisplatin solution was discarded at 30 days. All drug concentrations were taken from published IC_50_ concentrations relevant to each cell line, as demonstrated in Table 2.

### 2.2. Tissue Culture

Caov-3, a platinum-sensitive recurrent human ovarian cancer cell line, and Ovcar-3, a platinum-resistant human ovarian cancer cell line, were obtained from the American Type Culture Collection (Manassas, VA, USA). Caov-3 was grown in Dulbecco’s modified Eagle’s medium (DMEM; ThermoFisher Scientific, Waltham, MA, USA) with 10% FoundationTM fetal bovine serum (FBS; GeminiBio, West Sacramento, CA, USA) and 2% Gibco^®^ antibiotic–antimycotic (ThermoFisher Scientific). Ovcar-3 was grown in RPMI 1640 medium (ThermoFisher Scientific) with 10% FBS (GeminiBio), 2% Gibco^®^ antibiotic–antimycotic (ThermoFisher Scientific), and 10 µg/mL insulin (Sigma-Aldrich, St. Louis, MO, USA). Media were changed every 2–4 days and cells were passaged when they reached 80–90% confluence. Cells at passages 3–10 were used for experiments. To allow for direct comparison, cells grown in both 2D and 3D culture were simultaneously treated. All cells were incubated at 37 °C.

#### 2.2.1. Establishment of Concurrent 2D and 3D Tissue Culture

Caov-3 and Ovcar-3 were plated as single monolayers in triplicates in 6-well plates at 5 × 10^5^ cells per well. The following day, the cells were exposed to cisplatin, paclitaxel, cisplatin–paclitaxel, panobinostat, cisplatin–paclitaxel–panobinostat, SAHA and a DMSO control. Cells were imaged with an inverted microscope (Leica Microsystems, Buffalo Grove, IL, USA) and counted with ImageJ. Two-dimensional Caov-3 cells were counted on day 0, day 3, day 5, day 7, day 9 and day 12. Three-dimensional Ovcar-3 cells were counted on day 0, day 2, day 5 and day 7. Additionally, on the final day of experimentation, all cells were trypsinized with Trypsin-EDTA 0.25% (ThermoFischer Scientific) and counted directly with a Coulter Counter Z2 (Beckman Coulter, Brea, CA, USA).

The cells for 3D culture were cultured by the hanging-drop method [34] at a density of 1600 cells per drop for Caov-3 and 2500 cells per drop for Ovcar-3 in the appropriate growth media. The spheroids were then individually transferred into the wells of either 24- or 48-well bacterial plates and treated when the spheroid diameter reached 300–400 µm, which was 3–6 days following when they were initially plated in hanging drops. Individual spheroids were treated with cisplatin, paclitaxel, cisplatin–paclitaxel, panobinostat, cisplatin–paclitaxel–panobinostat, SAHA and a DMSO control. Three-dimensional cells were imaged at 2.5×, 5×, 10× and 20×, depending upon their size, with an inverted microscope (Leica Microsystems) and measured using ImageJ. Caov-3 spheroids were imaged and measured on day 0, day 3, day 5, day 7, day 9 and day 12. Ovcar-3 spheroids were imaged and measured on day 0, day 2, day 5 and day 7. Growth and cell count were monitored with both 2D and 3D culture for 7 days for Ovcar-3, and up to 14 days in Caov-3.

#### 2.2.2. Measurement of Spheroid Growth in 3D Culture

Direct geometric measurements have previously been effectively utilized to monitor spheroid growth [17]. Thus, we directly measured our spheroids after imaging them and followed their growth over time. ImageJ was used to measure the area of each spheroid and the average radius was extrapolated from this measurement. The formula:

A = πr^2^

was used to calculate the average radius, which was then used to calculate the volume of each individual spheroid at several time points as it progressed through the experiment. The formula:

V = 4/3πr^3^

was used to calculate the volume.

The percentage growth for each spheroid was measured relative to itself on day 0 and plotted using Prism8 with GraphPad. Percentage growth was measured with the following formula:

Percentage Growth = (current spheroid volume − spheroid volume on day 0)/(spheroid volume on day 0) × 100

As an additional metric, the fold change for each spheroid was also utilized. This was taken as the volume of the spheroid at a given point in time as relative to the volume of that same spheroid on day 0. This was also plotted using Prism8 with GraphPad. Fold changes were measured with the following formula:

Fold Change = current spheroid volume/spheroid volume on day 0.

### 2.3. Cellular Assays

#### 2.3.1. Apoptosis and Necrosis

The cells were treated under 3D conditions for 3 days. They were then subsequently treated according to the respective assay manufacturer’s instructions. The Invitrogen™ eBioscience™ Annexin V-FITC Apoptosis Detection Kit (ThermoFisher Scientific) was used to detect apoptosis, and the Invitrogen™ eBioscience™ Propidium Iodide Staining Solution (ThermoFisher Scientific) was used to detect necrosis. Three-dimensional cells were imaged at 2.5×, 5×, 10× and 20×, depending upon their size, with an inverted microscope (Leica Microsystems), utilizing both bright field and fluorescent microscopy.

#### 2.3.2. Viability, Cell Proliferation, Migration and Invasion Assays

A 3-(4,5-dimethylthiazol-2-yl) -2,5-diphenyltetrazolium bromide (MTT) cell proliferation assay kit was utilized, following the manufacturer’s recommendations (Invitrogen™ Vybrant™ MTT Cell Proliferation Assay Kit, ThermoFisher Scientific). Cellular material from previously treated spheroids was trypsinized and plated in 2D monolayer at 2 × 10^4^ cells/well in 6-well plates. Cells were counted prior to plating and the same number of viable cells were placed in each well.

The next day, cells were placed in standard growth media with different drug treatment conditions for 72 h. After MTT labeling, the 96-well cell plates were analyzed in the SpectraMax M3 (Molecular Devices, Sunnyvale, CA, USA) to obtain absorbance or optical density (OD) at 570 nm (and 540 nm for background reading). OD readings were then calculated by subtracting the background of 540 nm and the background absorbance of the media. These values were then compared to the control as a ratio of the cell viability at each treatment.

For the proliferation assays, cellular material from the previously treated spheroids was trypsinized and plated in 2D monolayer at 6000 cells/well in 96-well plates. Cells were counted prior to plating, and the same number of viable cells were placed in each well. Cells were plated in drug-free medium and monitored for proliferation over the course of 7 days. Metabolic activity was obtained by MTT, a 3-(4, 5-dimethylthiazol-2-yl)-2, 5-diphenyltetrazolium bromide cell proliferation assay kit (Molecular Probes, Eugene, OR, USA).

Cells derived from pretreated spheroids were additionally subjected to invasion and migration assays (CytoSelectTM 24-Well Cell Migration and Invasion Assay (8 µm, Colorimetric Format), Cat# CBA-100C, Cell Biolabs, San Diego, CA, USA). All assays were performed according to the manufacturer’s instructions.

#### 2.3.3. Observation of Colony Formation Following Treatment in 3D Culture

SFU colony formation unit assays were performed by adding 5690 cells from the trypsinized pre-treated spheroids to 100 mm plates (100 cells/mm^2^) and incubating for 14 days in the cell-appropriate media. Again, the same number of live cells was utilized per plate. At the end of 14 days, the media were removed, and the plates were washed with PBS. A 0.5% Crystal Violet staining solution (Sigma-Aldrich), which turns the cellular nuclei a deep purple color, was utilized to visualize the cancer colonies. The plates were incubated in 0.5% of Crystal Violet solution and subsequently washed 3 times in PBS prior to imaging. The cell colonies were imaged (by Apple iPhone 8, Cupertino, CA, USA). All pictures were taken 12 cm from the plates.

#### 2.3.4. Chemoresistance

Additional pre-treated spheroid-derived cells were plated in 96-well plates at 30,000 cells per well and retreated with classic chemotherapy (paclitaxel, cisplatin or cisplatin–paclitaxel) for evidence of chemoresistance over the course of 5 days. The same number of live cells was plated per well. Metabolic activity was obtained by MTT at the conclusion of 5 days.

### 2.4. Statistical Analysis

All experiments were repeated two to eight times. The number (*n*) of repeated experiments is listed in the figure legends (*n* = 3, *n* = 4, *n* = 5). Statistical analyses of viability were performed with unpaired t-tests. Statistical analyses of 2D cell counts as well as optical density differences in cellular proliferation, migration, invasion, and chemoresistance assays were completed with one-way analysis of variance (ANOVA) testing and post hoc testing (Tukey). ANOVA was utilized to analyze the differences in the percentage growth of 3D Caov-3 spheroids. Due to the distribution of the data, a Mann–Whitney U test was deemed more appropriate for the 3D Ovcar-3 spheroids. *p* < 0.05 was used for statistical significance. Data analysis was performed using SPSS Statistics software version 22 (IBM, Armonk, NY, USA).

## 3. Results

### 3.1. Spheroid Formation and Experimental Design

Three-dimensional culture is more clinically relevant than two-dimensional culture; therefore, an extended kinetic is more relevant than the typical shorter 24–72 h in which cells are monitored. Classic chemotherapy, cisplatin, paclitaxel, and cisplatin–paclitaxel are typically administered to patients every 3 weeks. It follows then that laboratory models should monitor these cells for longer periods of time. These classic chemotherapies were verified in 2D, animal models, and clinical trials; therefore, we wanted to compare the clinically relevant treatment in our 3D system. Additionally, we wanted to evaluate the efficacy of epigenetic therapy on ovarian cancer. Thus, we compare the classic approach to therapy with epigenetic therapy in a 3D model with an extended kinetic.

**Figure 1:**
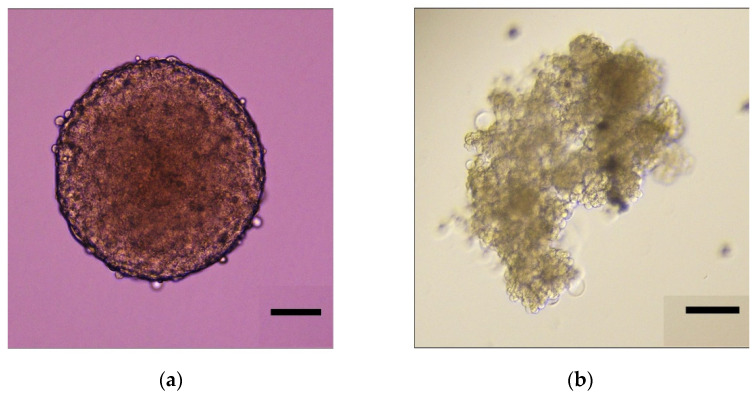
Representative images of 3D spheroid formation in ovarian cancer cell lines. Spheroids were grown until a diameter of ~400 µm. These images represent spheroids on day 0 prior to treatment with chemotherapy or epigenetic drugs. Scale bar is 100 µm. (**a**) Caov-3 spheroid; (**b**) Ovcar-3 spheroid.

We began by developing our tissue culture technique. We were able to make ovarian cancer spheroids that mimic those seen in patients using both the platinum-sensitive Caov-3 cell line and the platinum-resistant Ovcar-3 cell line, as described in the “Materials and Methods.” As evidenced in Figure 1a, Caov-3 in 3D culture forms uniform spheres that retain their shape over time. As seen in Figure 1b, Ovcar-3 in 3D culture forms an irregular structure with varying cell morphology that branches and clusters.

**Figure 2:**
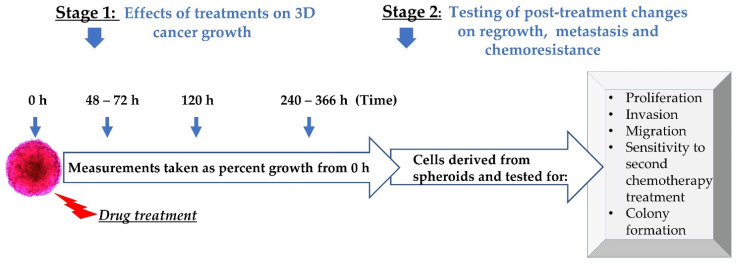
Experimental design. Following spheroid formation, epigenetic therapy or chemotherapy were administered in growth media. Measurements of spheroid growth were taken at specified time. Subsequently, 3D-derived cells from spheroids were evaluated for proliferation, invasion, migration, chemoresistance and colony formation.

As noted in day 0 in Figure 2, when the spheroids reached approximately 400 µm in diameter, both epigenetic and chemotherapeutic drugs were administered. Classic chemotherapeutic treatments included cisplatin, paclitaxel and cisplatin–paclitaxel (cis–tax). Epigenetic treatments included panobinostat and suberoylanilide hydroxamic acid (SAHA). Combination treatments included cisplatin–paclitaxel–panobinostat (cis–tax–pano) and cisplatin–paclitaxel–SAHA (cis–tax–SAHA). Spheroid growth was then followed for an extended kinetic, because this is more clinically relevant than shorter time periods that are traditionally observed. When the control spheroid for each experiment stopped growing, the experiment was concluded, which was on day 12 for Caov-3 and day 7 for Ovcar-3.

Several direct and indirect measurements were taken of our spheroids (radius, diameter, area and volume). In order to measure the change in our spheroids over time, percentage growth and fold changes were calculated in relation to each individual spheroid on day 0. Spheroid growth was measured as a function of volume. For each volume, the change in growth was noted for each spheroid relative to itself on day 0. The change in the growth of these spheroids was then monitored over a period of 2–14 days. To adequately compare 2D to 3D culture, this same timeline was applied to our 2D culture as well. At the conclusion of phase 1 of our process, spheroids were then trypsinized and spheroid-derived cells were tested for changes in different physiological parameters: cell growth, potency for migration, invasion, sensitivity to a second round of chemotherapy and cancer colony formation (CFU) (Figure 2).

### 3.2. In Caov-3 Cells, Paclitaxel Alone Does Not Suppress 3D Ovarian Cancer Growth in an Extended Kinetic Assay, Whereas Epigenetic Therapy Causes Spheroid Shrinkage

**Figure 3:**
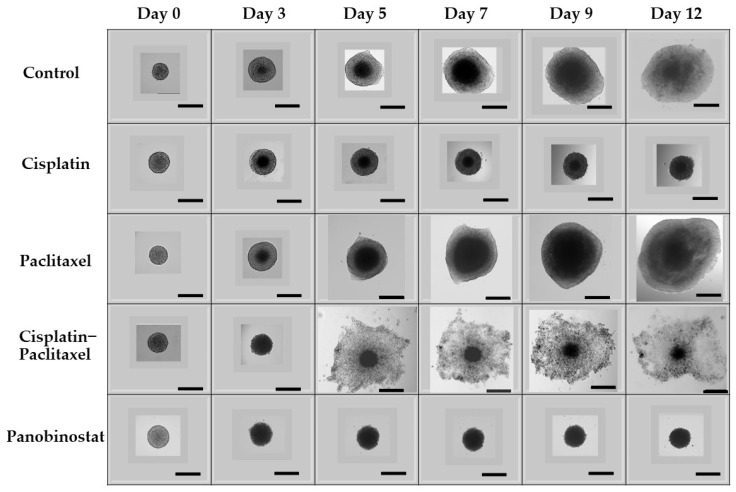
Direct visualization of 3D growth of Caov-3 cells under various treatments over time. In 3D culture, panobinostat is the only agent that causes Caov-3 spheroid shrinkage and decreases tumor spread. In contrast, in an extended kinetic, spheroids exposed to paclitaxel show extensive growth. Spheroids exposed to cisplatin and cisplatin–paclitaxel grew at a rate similar to the control. As seen in Appendix A, a similar trend was found with our Ovcar-3 spheroids. Scale bar is 500 µm (*n* = 3).

**Figure A1:**
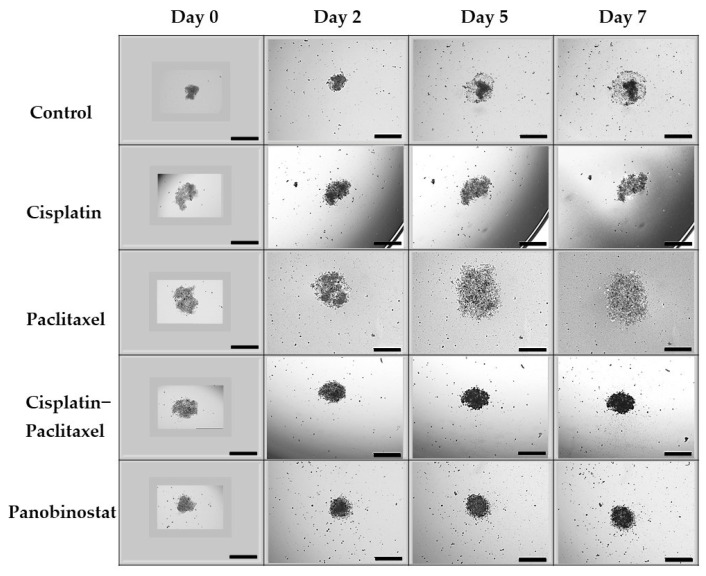
Direct visualization of 3D growth of Ovcar-3 cells under various treatments over time. In 3D culture, paclitaxel spheroids continue to grow to at the same rate of the control, while panobinostat is superior in effecting spheroid shrinkage. Spheroids exposed to cisplatin and cisplatin–paclitaxel grow at a rate similar to the control. Scale bar is 500 µm.

As visualized in Figure 3, epigenetic therapy induces spheroid shrinkage, whereas paclitaxel induces exponential growth in an extended kinetic. The effect of panobinostat alone on Caov-3 spheroids over an extended kinetic (12 days) is best visualized directly by phase contrast microscopy. Figure 3 shows that in this platinum-sensitive ovarian cancer cell line, when compared to the control, epigenetic therapy with panobinostat leads to both spheroid shrinkage and controls lateral spread of the tumor better than cisplatin. Panobinostat treatment had sustained cytotoxicity and shrinkage on Caov-3 spheroid growth in a short time kinetic as well as in an extended time kinetic. As compared to Caov-3 control spheroids, which grew 100-fold (10,000% growth), surprisingly, a single administration of panobinostat spheroids shrunk by 2-fold (up to −50% growth) (*p* < 0.0001) over the course of 12 days (Figure 3, microscopic images, and Figure 6, graphic representation). The trend in spheroid shrinkage and inhibition of spheroid expansion is visible across both experiments. The spheroids from cisplatin–paclitaxel (which is commonly utilized in clinical practice), dispersed many single live cells and small spheroids from the central spheroid (see Figure 3). This effect is not seen with other chemotherapy or epigenetic treatments. A similar trend was seen with the platinum-resistant Ovcar-3 spheroids. The schematic for this experiment can be found in Appendix A, Figure A1.

**Figure 4:**
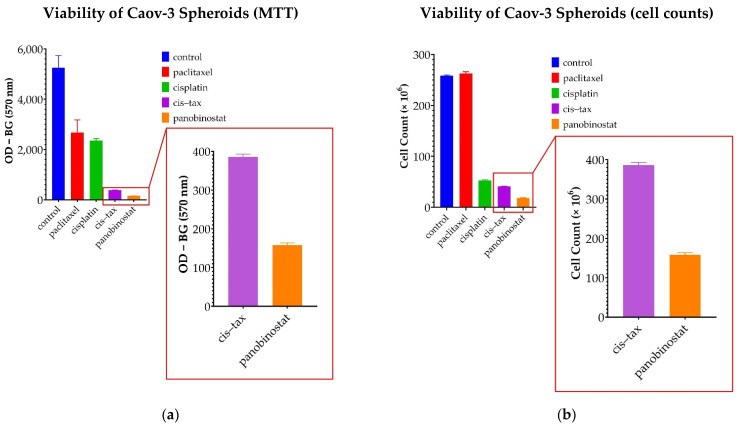
Viability assay of Caov-3 spheroids on day 3 following treatment. (**a**) In this MTT assay measuring the mitochondrial activity of the cells, optical density was used to represent the viability of the cells. Viability assays confirmed that panobinostat is more cytotoxic when compared to the control (*p* = 0.009) than either of the single agents, cisplatin (*p* = 0.03) or paclitaxel (*p* = 0.06). Although the classic combination of cisplatin–paclitaxel was effective in this assay when compared with the control (*p* = 0.01), the epigenetic therapy with panobinostat was twice as cytotoxic when compared directly to cisplatin–paclitaxel (*p* = 0.001) and over ten times as toxic as single-agent chemotherapy (cisplatin *p* = 0.003, paclitaxel *p* = 0.04) (*n* = 2) (**b**) Direct cell counts were utilized to confirm MTT findings. Paclitaxel was no more cytotoxic than the control (*p* = 0.278). All other treatments demonstrated cytotoxicity (*p* < 0.0001); furthermore, panobinostat was significantly more cytotoxic than all the other treatments (*p* < 0.001) (*n* = 2).

To confirm these observations, viability assays were performed on Caov-3 spheroids following treatment. As seen in Figure 4a, optical density, measured as OD − BG (at 570 nm), was utilized for the MTT assay. Optical density for the control cells was measured at 5253. Cells exposed to panobinostat were less viable than the control at an optical density of 158.2 (*p* = 0.009). However, the optical density for cells exposed to the commonly utilized clinical treatment cisplatin–paclitaxel was 385.9, which is statistically less viable than the control (*p* = 0.01), but still more viable than panobinostat (*p* = 0.001). When classic chemotherapy was utilized with single agents, the optical density was measured at 2679 for paclitaxel, which was not statistically significant when compared to the control (*p* = 0.066), and 2360 for cisplatin, which was statistically less significant than the control (*p* = 0.03). Taken together, in these assays, the treatment with panobinostat was found to be more than 2 times as cytotoxic as cisplatin–paclitaxel (*p* = 0.001) and 10 times more cytotoxic than either agent alone (cisplatin *p* = 0.003, paclitaxel *p* = 0.04).

Direct cell counts were used to confirm these findings, as seen in Figure 4b. When cell counts were measured, the control was not significantly different from cells exposed to panobinostat (*p* = 0.278). Cell counts for all other treatments, cisplatin, paclitaxel, cisplatin-paclitaxel and panobinostat were significantly lower than the control (*p* < 0.0001). Additionally, cell counts for panobinostat were significantly less than those cells exposed to cisplatin and paclitaxel as single agents, as well as cells exposed to the combination of cisplatin–paclitaxel (*p* < 0.0001).

**Figure 5:**
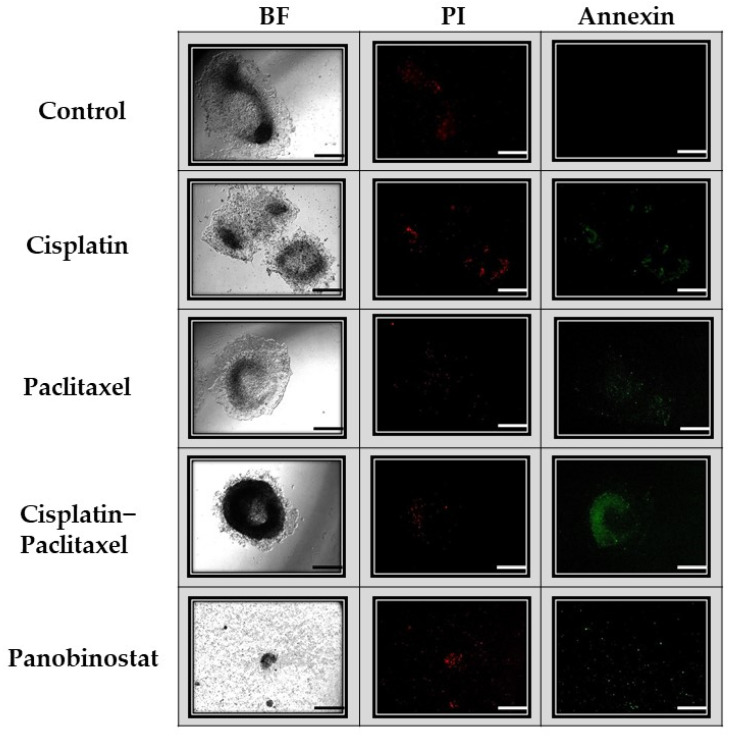
Visual display of apoptosis and necrosis of Caov-3 spheroids on day 7. Caov-3 spheroids were treated and imaged on day 7 with green annexin V staining (apoptosis) and red propidium iodide (PI) staining (necrosis), as well as under bright field microscopy (BF). Under control conditions, after 7 days, the spheroids showed little necrosis and no apoptosis. Spheroids exposed to single-agent chemotherapy (cisplatin alone, paclitaxel alone) demonstrated the same low level of apoptosis and necrosis as the control. Those spheroids exposed to cisplatin–paclitaxel demonstrated the brightest apoptosis staining and little necrosis staining. Spheroids treated with panobinostat had little cellular material left due to the cytotoxicity of the drug; however, what is present demonstrates high levels of apoptosis and necrosis. Scale bar is 100 µm (*n* = 2).

Similarly, apoptosis, necrosis and viability assays further demonstrate the superiority of panobinostat. As seen in Figure 5, panobinostat affects not only spheroid shrinkage and dispersion, but also necrosis (red fluorescence) and apoptosis (green fluorescence). In Figure 5, the control spheroid, as seen on day 7, demonstrates minimal necrosis, but no apoptosis. The spheroid exposed to paclitaxel displays a similar level of necrosis as the control, but slightly more apoptosis. The cisplatin spheroids display still more apoptosis and necrosis, whereas the spheroid exposed to cisplatin–paclitaxel demonstrates a greater level of apoptosis than necrosis. Very few cells were visible on day 7 when spheroids were exposed to panobinostat; however, those present demonstrated a high level of staining for both apoptosis and necrosis.

**Figure 6:**
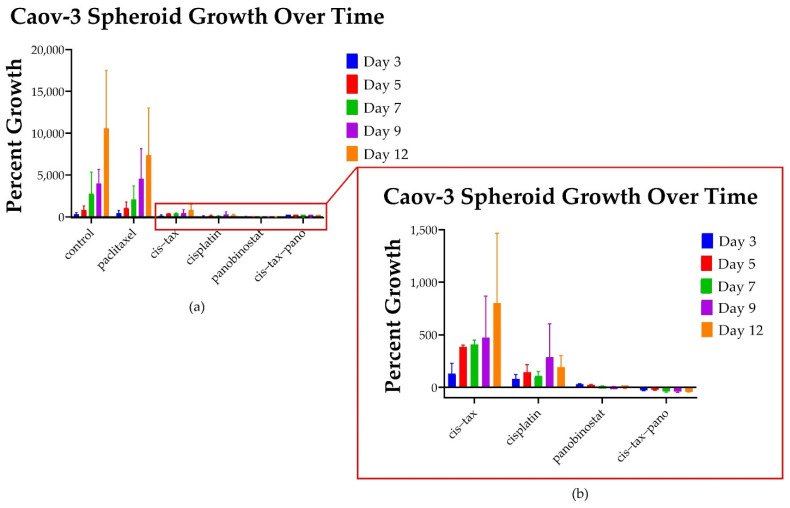
Graphic representation of the 3D growth of platinum-sensitive spheroids over time. (**a**) Spheroids exposed to paclitaxel on day 3 exceeded that of the control: Caov-3 spheroids demonstrated 293% growth (3-fold) under control conditions and 427% growth (4-fold) with paclitaxel alone (*p* < 0.0001). However, the control spheroids grow to 10,000% (100-fold) their original size on day 12, whereas spheroids exposed to paclitaxel grow to 427% (4-fold) their original size. Spheroids exposed to cisplatin alone grow about 200% (2-fold) their original size. (**b**) The standard treatment, cis-tax, is more effective than paclitaxel alone, but still not as effective as the epigenetic therapy panobinostat, and especially in combination with standard chemotherapy. More effective than chemotherapy, the epigenetic therapy panobinostat not only curbed growth, but affected spheroid shrinkage, by 6% (*p* < 0.0001) for panobinostat alone and 42% (*p* < 0.0001) for the combination of cis–tax–pano (*p* < 0.0001) (*n* = 3).

**Figure 7:**
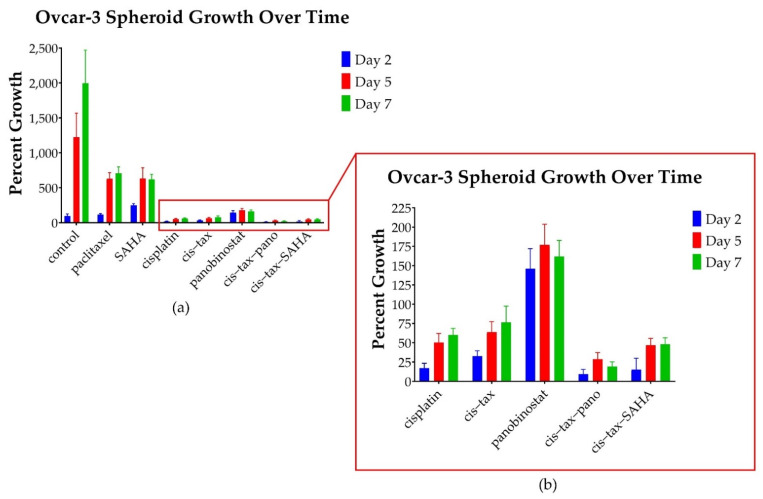
Percentage growth of cisplatin-resistant Ovcar-3 spheroids at selected timepoints under various treatments. (**a**) Control spheroids grow to 1994% their original size, whereas those exposed to paclitaxel and SAHA grow to 706% and 620% of their original size (*p* = 0.01) However, other treatments are far more effective. No treatments affect spheroid shrinkage. (**b**) Spheroids exposed to panobinostat grew to 162% of their original size (*p* = 0.01), whereas, those exposed to the combination of cisplatin–paclitaxel-panobinostat grew to 19% of their original size (*p* = 0.01) and those exposed to the combination of cisplatin–paclitaxel-SAHA grew to 48% of their original size (*p* = 0.029). Spheroids exposed to cisplatin grew to 60% of their original size (*p* = 0.016). The spheroids exposed to the combination of cis–tax grew to 76% their original size (*p* = 0.29) (*n* = 4).

Throughout Phase 1 of our experiment (Figure 2), the radius, diameter, area and volume of each individual spheroid was measured relative to itself. As such, differences in percentage growth from day zero confirm the observations that taxol-based therapy induces exponential 3D ovarian cancer growth, whereas epigenetic therapy results in sustained cytotoxicity. As seen in Figure 6 and Figure 7, we then evaluated how the single agents, cisplatin and paclitaxel, compared directly to single-agent epigenetic therapies and combinations of classic chemotherapy with epigenetic therapy relative to the control. Unexpectedly, ovarian cancer spheroids treated with paclitaxel alone exhibited exponential 3D growth that was similar to the growth observed in the control. Relative to their size on day 0 (the start of treatment), on day 3, the platinum-sensitive Caov-3 spheroids demonstrated 293% growth (threefold) when untreated (control; Figure 6), and 427% growth (fourfold) with paclitaxel alone (*p* < 0.0001). In comparison, spheroids exposed to cisplatin grew 80% (0.8-fold) (*p* < 0.0001) and 130% (1.3-fold) with cis–tax (*p* < 0.0001) when compared to the control. In contrast, on day 3, spheroids exposed to panobinostat grew only by 30% (0.3-fold) for panobinostat alone (*p* < 0.0001), and had shrunk by 30% (0.3-fold) for the combination of cis–tax–pano (*p* < 0.0001) when compared to the control.

By day 12, paclitaxel Caov-3 spheroids grew by 7500% (75-fold), whereas control spheroids treated with DMSO had expanded by 10,000% (109-fold) (*p* < 0.0001). Spheroids treated with cisplatin alone expanded to 192% (twofold) (*p* < 0.0001). Moreover, spheroids treated with combined chemotherapy, cisplatin and paclitaxel, demonstrated shrinkage of the central spheroid’s size, but when lateral spread and single cell seeding was accounted for, spheroids still grew 800% (80-fold) on day 12 (*p* < 0.0001) (Figure 6). This result was surprising, because patients who are platinum-sensitive (which is represented by the cell line Caov-3) are usually treated with a platinum agent when they recur. In contrast, cells exposed to panobinostat not only prevented spheroid growth, but shrank by 6% (*p* < 0.0001), and those exposed to cis–tax–pano shrank by 42% (*p* < 0.0001).

### 3.3. In Ovcar-3 Cells, the Combination of Classic Chemotherapy with Epigenetic Therapy Is Most Effective

In contrast to platinum-sensitive patients, platinum-resistant patients are not retreated with a platinum agent. In fact, these patients are very difficult to successfully treat with any currently available treatment. Not surprisingly, no therapies resulted in spheroid shrinkage of our platinum-resistant Ovcar-3 spheroids. As seen in Figure 7, all therapies had a partial effect in halting spheroid growth with the combination of cisplatin–paclitaxel, with epigenetic therapy being most effective.

Relative to the control, on day 2, Ovcar-3 spheroids responded the best to the combinations of the classic chemotherapy cisplatin–paclitaxel with the epigenetic therapies panobinostat or SAHA. For example, spheroids exposed to cis–tax–pano grew only 9.5% (*p* = 0.01) and those exposed to cis–tax–SAHA grew only 15% (*p* = 0.05), whereas cisplatin grew 17% (*p* = 0.016) and cis–tax grew 32% (*p* = 0.05). Platinum-resistant Ovcar-3 spheroids grew beyond the level of the control (96% growth) with the treatments: paclitaxel (117%, *p* = 0.352), and panobinostat (146%, *p* = 0.171), but these values were not statistically significant. On day 2, SAHA alone had induced growth beyond the level of the control (250% growth, *p* = 0.010).

By day 7, however, the control had grown to 1994% of its original size, whereas SAHA had only grown to 620% of its original size (*p* = 0.01). Epigenetic therapies in combination with classic chemotherapy were the most cytotoxic over time, as Ovcar-3 spheroids exposed to cis–tax–pano exhibited 19% growth (*p* = 0.01) and cis–tax–SAHA exhibited 48% growth (*p* = 0.029). Spheroids exposed to paclitaxel demonstrated 706% growth (*p* = 0.01), whereas spheroids exposed to panobinostat demonstrated 162% growth (*p* = 0.01) and those exposed to cisplatin alone demonstrated 60% growth (*p* = 0.016). Ovcar-3 spheroids exposed to cisplatin–paclitaxel demonstrated 76% growth when compared to their original size, but this was not statistically significant (*p* = 0.29).

### 3.4. Cells Observed over an Extended Kinetic in 2D Culture Demonstrate the Same Trends Seen in 3D Culture, However, the Effect Is More Pronounced in 3D as Compared to 2D

**Figure 8:**
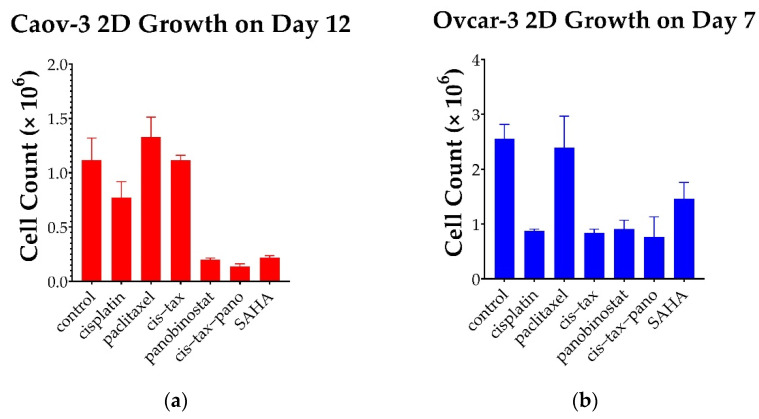
Growth of recurrent HGSC in 2D over a prolonged kinetic. (**a**) Following treatment with classic chemotherapy (cisplatin, paclitaxel, cis–tax), Caov-3 cells rebounded within 12 days and regrew to the level of the control. In contrast, sustained cytotoxicity was noted with epigenetic therapy (panobinostat, cis–tax–SAHA and SAHA), where cell counts were diminished by 74–80% (*p* < 0.001). (**b**) Ovcar-3 cells recovered over the course of 7 days in response to paclitaxel; however, other treatments diminished cell counts by one-third to one-half (*p* < 0.001) (*n* = 3).

Initially, and surprisingly, 3D Caov-3 and Ovcar-3 cells experience exponential growth within 48–72 h of paclitaxel administration as single agents, and this is sustained throughout the course of the experiment (Figure 6 and Figure 7). In stage 1 of our experiment (Figure 2), a simultaneous 2D procedure was undertaken to directly compare the results of our study in 2D and 3D. For the 2D portion of our experiment we imaged and directly counted cells throughout the 12-day experiment for Caov-3 and 7-day experiment for Ovcar-3. Paclitaxel follows this same trend at the conclusion of our 2D experiment as in our 3D experiment, but the result is not as dramatic. As seen in Figure 6, In 3D on day 12, Caov-3 spheroids grew 103-fold in the presence of paclitaxel, whereas the control grew 109-fold (*p* = 0.4). In comparison, as seen in Figure 8a, in 2D monolayer on day 12, Caov-3 grew 2-fold in the presence of paclitaxel, whereas the control grew 1.69-fold (*p* = 0.375).

Caov-3 cells in 2D grow to about the level of the control with classic chemotherapy. In response to cisplatin alone and cis–tax, cells grow 1-fold at the conclusion of 12 days, while the control grows 1.69-fold (*p* = 0.05, *p* = 1.0) (Figure 8a). This, again, is a contrast to Caov-3 cells in 3D which grew 109-fold under control conditions, 95-fold in response to cis–tax and 2-fold in response to cisplatin alone, (*p* < 0.0001) (Figure 6). Ovarian cancer cells continue to grow over an extended kinetic with classic chemotherapy, but epigenetic therapy retains cytotoxicity over time as compared to the control.

In response to the epigenetic therapies, Caov-3 cell counts on day 12 in 2d were diminished by 74% with SAHA (*p* < 0.001), 76% with panobinostat alone (*p* < 0.001) and 83% in response to cis–tax–pano (*p* < 0.001) (Figure 8a). By comparison, Caov-3 spheroids shrunk by 35% (*p* < 0.0001) and those exposed to cis–tax–pano shrunk by 29% (*p* < 0.0001) as compared to the control (Figure 6).

As seen in Figure 8b, Ovcar-3 cells in 2D monolayer on day 7 demonstrated twofold growth in response to both paclitaxel and the control (*p* = 0.989). Cisplatin diminished cell counts by 37% (*p* < 0.001) and cis–tax diminished cell counts by 45% (*p* < 0.001) in our Ovcar-3 2D model. Panobinostat and cis–tax–pano cut cell counts by 38% and 39%, respectively (*p* < 0.001), whereas SAHA diminished cell counts by half as compared to the control (*p* = 0.014) (Figure 8b).

### 3.5. Physiologic Changes in Ovarian Cancer Cells Following Chemotherapy or Epigenetic Treatments in 3D Culture

We then sought to analyze different properties of our 3D-cultured cells. Cells in 3D culture are organized differently from those in 2D culture [16]. As such, 3D spheroids have different cell–cell contacts and extracellular matrices [16]. These bundles of cells can differentiate, secreting different growth factors and generating their own secretome [16]. Thus, we designed the following experiments to evaluate differences in our spheroid-derived cells, not only as a result of their different treatments and prolonged kinetic, but also because of the different structures in which they were treated. In this stage, we also analyzed for physiologic changes in these cells. Ovarian cancer cells in patients can develop chemoresistance after one or two cycles; therefore, we hypothesized that ovarian cancer cells in 3D culture might change their potency for proliferation, invasion, migration and colony formation following treatment. In this way, 3D culture might address the discrepancy between what is observed in 2D cultures and the chemoresistance that is seen clinically in patients.

#### 3.5.1. Spheroid-Derived Cells Exhibit No Change in Proliferation Following Treatment with Paclitaxel; However, There Is a Decreased Proliferative Ability Noted following Treatment with Epigenetic Therapy

**Figure 9:**
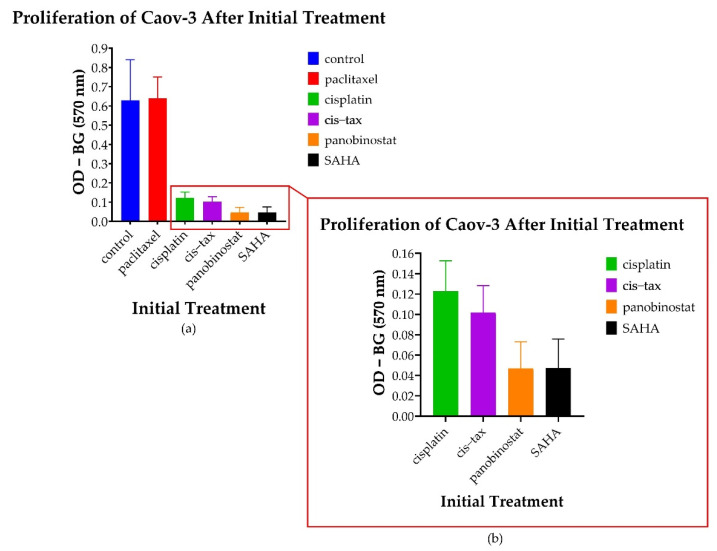
Changes in the proliferation of 3D-derived cells following various treatments. Cells derived from Caov-3 spheroids pretreated with a DMSO control, paclitaxel, cisplatin, cisplatin–paclitaxel and panobinostat were allowed to regrow for 7 days. Optical density (OD) was measured at 570 nm minus the background (BG). (**a**) Cells treated with paclitaxel demonstrated regrowth almost to the level of the control (*p* < 0.001). (**b**) Cells pretreated with panobinostat and SAHA demonstrated the sustained inhibition of proliferation of Caov-3 spheroid-derived cells as compared to the control (*p* < 0.001) (*n* = 8).

After cell growth was monitored over a prolonged kinetic in both 2D and 3D models, we then sought to evaluate the metastatic potential of our 3D cells. This was performed with MTT assays (measured with optical density and calculated as OD-BG at 570 nm) as well as colony formation. As evidenced in Figure 9, Caov-3 cells that were initially treated with classic chemotherapy in spheroid form had greater proliferative potential following exposure to paclitaxel than any other treatment (*p* < 0.001). However, the cells derived from Caov-3 spheroids initially treated with the epigenetic therapies panobinostat and SAHA exhibited markedly diminished proliferative potential (*p* < 0.001). Cells treated with cisplatin alone and cisplatin–paclitaxel also exhibited diminished proliferation (*p* < 0.001). Additionally, we observed a trend where panobinostat and SAHA were more effective in reducing proliferation than cisplatin and cisplatin–paclitaxel; however, this did not reach statistical significance (*p* = 0.204 to *p* = 0.659).

#### 3.5.2. Cells Treated with Paclitaxel Demonstrate Increased Migration Capability, Whereas Those Treated with Epigenetic Therapy Demonstrate Decreased Invasion Capability

**Figure 10:**
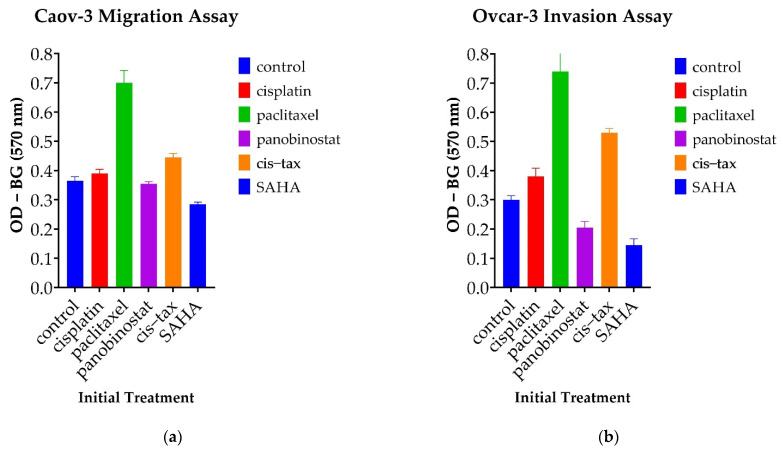
Changes in migration and invasion capacity in spheroid-derived cells following their initial treatment in a 3D system. Surprisingly, cells from both lines exposed to paclitaxel demonstrated a greater ability to migrate and invade than the control. Optical density was measured at 570 nm minus the background. (**a**) Pretreated Caov-3 cells from spheroids were replated over migration assays. Cells treated with epigenetic therapy had diminished propensity for migration, although these did not reach statistical significance. (**b**) Pretreated Ovcar-3 cells from spheroids were replated over invasion assays. Again, epigenetic therapy appeared to thwart the invasion capability of these cells, although they did not reach statistical significance (*n* = 2).

Recurrent HGSC notoriously migrates and invades both locally and distally within patients; therefore, we next tested changes in the ability of our cells to migrate and invade. As shown in Figure 10, spheroid-derived ovarian cancer cells had differing invasion and migration abilities based on their initial treatment in our 3D environment. Again, spheroid-derived cells initially treated with the classic chemotherapy and paclitaxel alone demonstrated increased migration in Caov-3 and invasion in Ovcar-3. Interestingly, this capability was significantly higher than that of the control (*p* < 0.001) and persisted across both our platinum-sensitive and platinum-resistant cell lines.

Caov-3 spheroid-derived cells previously exposed to the epigenetic therapies of panobinostat and SAHA both displayed a decreased propensity for migration when compared to the control; however, only the treatment of SAHA reached statistical significance (*p* = 0.005). Ovcar-3 cells previously treated in spheroid form with paclitaxel and cisplatin–paclitaxel displayed an increased propensity for invasion over the control (*p* > 0.001 and *p* = 0.015, respectively). There was a trend toward decreased invasion with the epigenetic therapies panobinostat and SAHA; however, these optical densities did not reach statistical significance (*p* = 0.879 and *p* = 0.086). Interestingly, in our Ovcar-3 invasion assays, SAHA outperformed all three classic chemotherapeutic regimens, paclitaxel, cisplatin and cisplatin–paclitaxel (*p* < 0.001, *p* = 0.014, and *p* = 0.001, respectively). The results from our Caov-3 invasion assay were not significant (data not shown), highlighting the fact that different cell lines within ovarian cancer harness their metastatic potential with differing mechanisms.

#### 3.5.3. Epigenetic Therapy Reduces the Ability for Ovarian Cancer Cells to Form Colonies, Whereas Classic Chemotherapy Does Not Have This Effect

**Figure 11:**
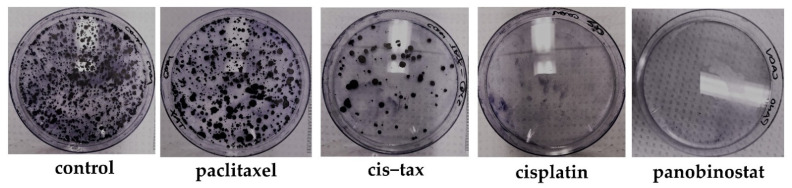
Cancer colony formation of Caov-3 cells derived from spheroids following initial treatment in a 3D environment. One hallmark of aggressive recurrent cancer is the potential formation of cancer cell colonies, because these cells have the unique ability to seed and colonize a new environment. Therefore, we replated Caov-3 spheroid-derived cells previously treated with a DMSO control, paclitaxel, cisplatin, cis–tax and panobinostat. Cells previously exposed to paclitaxel formed colonies with a density similar to the control. Cells exposed to cisplatin and cis–tax grew substantially fewer colonies, whereas no colonies are visible with cells treated with panobinostat (*n* = 3).

A known characteristic of HGSC is its ability to seed the peritoneal cavity causing micrometastases. Therefore, we analyzed the ability or inability of 3D derived ovarian cancer cells to reform colonies following treatment with either classic chemotherapy or epigenetic therapy. As evidenced in Figure 11, spheroid-derived cells previously treated with paclitaxel form colonies that are a similar density to those of the control. There are sequentially fewer colonies formed from cells derived from spheroids previously treated with cisplatin–paclitaxel and cisplatin. No visible colonies were seen in spheroids previously treated with panobinostat.

### 3.6. Chemoresistance Observed in 3D Ovarian Cancer Cells Is Reversible with Epigenetic Therapy

Moving forward, we analyzed the selectivity of ovarian cancer to a second round of chemotherapy after the first round of treatments was given in the 3D model. For this purpose, we derived the ovarian cancer cells from the spheroids after they had been allowed to grow in treated and untreated (DMSO control) media for 14 days (Caov-3) or 7 days (Ovcar-3). Subsequently, the derived cells were treated again with a DMSO control or classic chemotherapy (paclitaxel, cisplatin or cisplatin–paclitaxel). The survival ability of the cells was quantified by MTT viability assay, which was measured in optical density.

**Figure 12:**
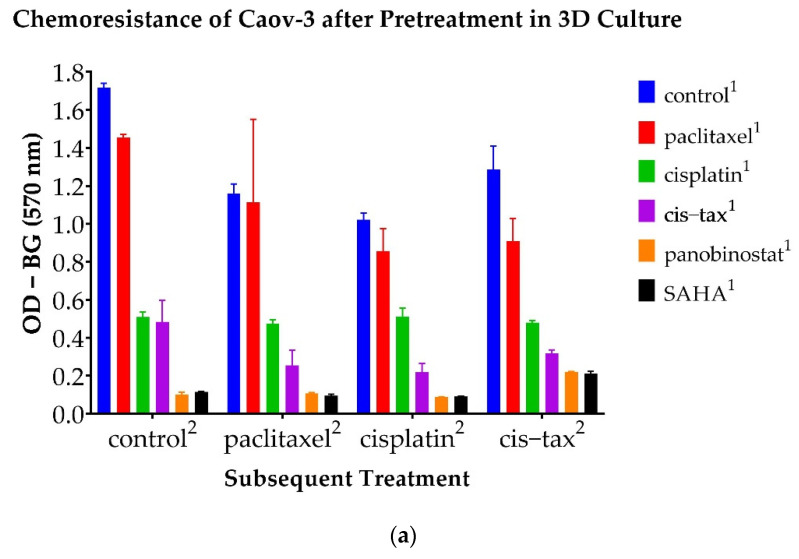

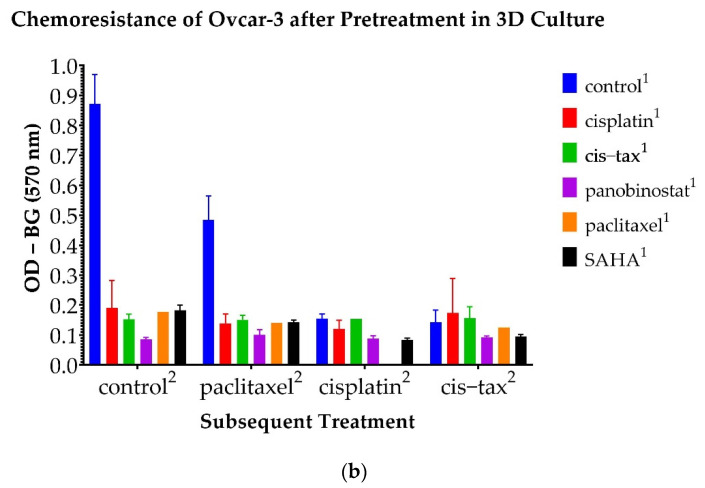
Optical density of ovarian cancer cells following a second round of classic chemotherapy. Spheroids were pretreated with a DMSO control, paclitaxel, cisplatin, cisplatin–paclitaxel, panobinostat and SAHA. They were then collected following primary treatment (denoted with the superscript “1”) and retreated with a subsequent treatment (denoted with the superscript “2”). Optical density was measured at 570 nm minus the background. (**a**) Caov-3 cells pretreated with panobinostat and SAHA demonstrated sustained chemosensitivity when retreated with classic chemotherapy (*p* < 0.001). (**b**) All Ovcar-3 cells were somewhat resensitized to chemotherapy after one treatment; however, panobinostat and SAHA were the most effective in reversing chemoresistance (*p* < 0.001) (*n* = 2).

Caov-3 cells are known to be cisplatin-sensitive. As seen in Figure 12a, cells derived from Caov-3 spheroids subsequently treated with cisplatin were reduced by 50% when they were previously exposed to cisplatin (*p* < 0.001), and by 80% when they were previously exposed to cis–tax (*p* < 0.001). Interestingly, cells previously treated with paclitaxel and subsequently treated with cisplatin became platinum-resistant, because they had a similar density to the control (*p* = 0.159). The epigenetic therapies panobinostat and SAHA when used prior to cisplatin most significantly sensitized cells to platinum, because only 9% remained following these treatments (<0.001).

Similar trends were seen in the Caov-3 line with another regiment commonly utilized in clinical practice, cisplatin–paclitaxel (Figure 12a). Caov-3 cells previously treated as spheroids with paclitaxel retained 70% of their cellular material when subsequently exposed to cis–tax (*p* = 0.013), and those previously treated with cisplatin retained 37% of their material (*p* < 0.001). However, those previously treated with cis–tax kept 25% of their original cellular material when retreated with cis–tax (*p* < 0.001). Cells previously retreated with the epigenetic therapies panobinostat and SAHA diminished growth in cells subsequently treated with cis–tax by 17% and 16%, respectively (*p* < 0.001), indicating reduced chemoresistance and increased chemosensitivity with epigenetic therapies.

Cells derived from Caov-3 cells previously treated with paclitaxel were resistant to paclitaxel after only one round of chemotherapy because they also grew to the level of the control (*p* = 1.0) (Figure 12a). Cells previously treated with cisplatin and subsequently treated with paclitaxel grew to 48% of the control, although this was not statistically significant (*p* = 0.064). Cells previously treated with cis–tax and subsequently treated with paclitaxel retained 25% of their cellular material (*p* = 0.019). Cells previously treated with panobinostat and SAHA were most effective at maintaining chemosensitivity when retreated with paclitaxel, because those cells grew to 10% of the control (*p* = 0.008 and *p* = 0.009, respectively).

Furthermore, Caov-3 cells first treated with panobinostat and SAHA demonstrated sustained cytotoxicity, because they failed to regrow even under control conditions. As evidenced in Figure 12a, regardless of the retreatment, spheroids first treated with panobinostat and SAHA developed no chemoresistant properties, and were more susceptible to classic chemotherapy, paclitaxel, cisplatin and cisplatin–paclitaxel. In contrast, ovarian cancer cells that had been exposed to classic chemotherapeutic agents first demonstrated more chemoresistance in the second cycle of chemotherapy.

As seen in Figure 12b, the epigenetic drugs panobinostat and SAHA were also effective in reducing chemosensitivity in the Ovcar-3 cell line; however, this effect was not as pronounced as in the Caov-3 cell line. This line is platinum-resistant; therefore, cells previously exposed to cisplatin grew to the level of the control when exposed to all other chemotherapies in the second round: cisplatin, cis–tax and paclitaxel (*p* = 0.392). However, cells previously exposed to panobinostat and SAHA grew to densities that were 58% and 53% of the control, respectively (*p* = 0.005 and *p* = 0.003). Thus, epigenetic therapy has the ability to reverse platinum resistance in this known platinum-resistant cell line.

Interestingly, as with the Caov-3 cell line, Ovcar-3 cells previously treated with paclitaxel retained nearly all of their cellular material when re-exposed to paclitaxel, indicating not only platinum resistance, but taxane resistance as well (Figure 12b). All other pre-treatments, cisplatin, cis–tax, panobinostat and SAHA, retained 20–30% of their cellular material when given paclitaxel in a second round of chemotherapy (*p* < 0.001).

As seen in Figure 12b, Ovcar-3 cells previously treated with paclitaxel, cisplatin and cis–tax retained all of their cellular material when retreated with cis–tax, again indicating both platinum and taxane resistance.

## 4. Discussion

Current ovarian cancer treatments fail in platinum-sensitive and platinum-resistant patients due to rapidly developing chemoresistance. The lethality of ovarian cancer lies not in its initial diagnosis, but in its recurrence, where treatment is rarely curative. Chemoresistant clones, which are often present in the initial tumor, grow selectively under the pressure of chemotherapy [35,36]. Furthermore, there is evidence that patients with tumors with high clonal expansion of these stem cells have shorter disease-free intervals and shorter survival [35]. Mutational profiling of recurrent disease has identified extensive intertumoral heterogeneity and genomic instability, which allows for the formation of these chemoresistant clones [25,37,38].

The cell lines Caov-3 and Ovcar-3 have traditionally been used to study platinum-sensitive and platinum-resistant ovarian cancer, respectively. However, it must be noted that these terms are in reference to how patients respond to platinum-based chemotherapy, and not necessarily how cells respond to these treatments in vitro. Ovarian cancer patients that recur over 6 months following the completion of chemotherapy are platinum-sensitive, whereas ovarian cancer patients that recur within 6 months of completion of chemotherapy are platinum-resistant [5]. Although our Ovcar-3 cells did respond to cisplatin in our 7-day experiments (Figure 7), this did reflect what is typically seen in platinum-resistant patients. Women with platinum-resistant ovarian cancer do enter remission following treatment with cisplatin, but then they recur within 6 months of treatment.

Traditional 2D cell culture is unable to adequately study the chemoresistant clones associated with both platinum-sensitive and platinum-resistant disease because these chemoresistant cells do not proliferate in 2D; they instead grow in 3D spheroids in patients [39]. Although the trend in classic chemotherapy outcomes is similar between 2D and 3D systems over a short period of time, overall, drug sensitivity and final treatment outcomes over a prolonged time period can be very different. This may directly explain the difference that we observe in disease progression in patients versus traditional 2D culture models and point to the superiority of a 3D model in studying ovarian cancer in vitro.

Two-dimensional systems are certainly advantageous because they are timely and efficient, but the results can be misleading [14]. The disadvantage of 3D systems is that the process is slow due to the high level of difficulty in 3D culture setup, maintenance and the interpretation of results. Despite these disadvantages, we think that the adoption of 3D systems is critical for the development of novel, effective cancer treatments. Cell–cell interactions, the extracellular matrix, the tumor microenvironment, growth factor secretion, and cancer stem cells are all different in 3D as opposed to 2D culture [40,41]. There is evidence that HGSC can transition from an epithelial to a mesenchymal subtype and that this subtype leads to platinum resistance [42]. Ovarian cancer cells undergoing this transition loosen their cell–cell contacts, acquiring stem cell characteristics and advanced metastatic potential [43]. These cell–cell interactions are likely better captured in 3D. Ovarian cancer cells that are cultured in 3D often express different adherens junction proteins and are often more chemoresistant than the same cells that are cultured in 2D monolayers [10]. Furthermore, the prominent mechanism of HGSC is the direct metastasis of 3D spheroids throughout the abdominal cavity via ascites [11]. These spheroids often contain cancer stem cells, which are also known as chemoresistant clones [38,43]. Hence, it appears that 3D models are likely the most clinically relevant way of studying chemoresistant ovarian cancer cell lines [14].

One unique property of our 3D spheroid system is our ability to directly compare the growth of 3D spheroids between different treatments for prolonged periods of time (Figure 3). This may be critically important because more complete upfront eradication may confer a survival benefit. The longer cancer cells are exposed to chemotherapy, the more chemoresistance is acquired. Moreover, we followed the outcomes of classic chemotherapy and epigenetic drug therapies in two weeks, instead of few days. When classic chemotherapy is utilized to treat HGSC, it is most commonly performed in 21-day cycles. Thus, we believe that preclinical studies should also be conducted over longer periods of time. The long-term kinetic of our experiment is designed to monitor the long-term efficacy of classic chemotherapy and epigenetic therapy regarding recurrent disease.

Our data show significant differences from classic ovarian cancer treatments; therefore, we believe that a 3D tissue culture system is absolutely necessary in developing novel cancer treatments because our results show that the same set of drugs used in 2D versus 3D tissue culture may lead to different and sometimes completely unexpected long-term outcomes (Figure 3 and Figure 8). The most surprising result of our study was that one administration of a single paclitaxel agent could cause the exponential growth of Caov-3 and Ovcar-3 cells over an extended kinetic in a 3D model. Even in the presence of known platinum sensitivity, classic single-agent chemotherapy did not appear to confer a benefit (Figure 6 and Figure 7). Furthermore, our model demonstrates that classic chemotherapy induces cell death mostly by apoptosis in our spheroid model, whereas epigenetic therapy induces cell death by both apoptosis and necrosis (Figure 5). These findings of cytotoxicity were confirmed with our viability assay (Figure 4). Additionally, epigenetic therapy was twice as effective against Caov-3 as the classically used cisplatin–paclitaxel (Figure 4).

A single administration of the epigenetic drug panobinostat was directly cytotoxic over 1–2 weeks across both cell lines and in both 2D and 3D tissue culture models (Figure 9). SAHA was highly effective in 2D, but had mixed results in 3D (Figure 6, Figure 7 and Figure 8). SAHA and panobinostat both work as histone deacetylase (HDAC) inhibitors, preventing the unwinding of DNA and subsequent DNA expression. Due to the high level of tumor heterogeneity found in HGSC, different epigenetic therapies can have different effects on tumor proliferation. We believe that intertumoral heterogeneity is responsible for our unexpected results, namely, the exponential growth of our cell lines in the presence of classic chemotherapy and the inconsistent results we encountered with SAHA. As others have identified, the efficacy of epigenetic therapy and its synergism with classic chemotherapy is dependent upon the cell line and tumor-specific characteristics, which again are attributed to the wide tumor heterogeneity encountered in HGSC [44].

The classic model of experimentation starting with 2D in vitro studies, then animal in vivo projects, and finally to clinical trials has not been as effective in predicting how HGSC will respond to epigenetic therapy. For example, SAHA was found to induce tumor suppressor genes, apoptosis and cell cycle arrest in ovarian cancer cell lines and xenografts with nude mice [45]; however, a phase II study of the drug vorinostat demonstrated minimal activity in recurrent platinum refractory HGSC patients [46]. Furthermore, we believe that our 3D model and extended kinetic more closely mimic the behavior of these spheroids in vivo; thus, we experienced similar results to the phase II study demonstrating the limited efficacy of vorinostat in vivo [46]. Other trials with SAHA, such as the one performed by Matulonis et al., demonstrated that when used in direct combination with carboplatin and gemcitabine, vorinostat was effective in 16 patients, but too toxic to tolerate [47].

Epigenetic therapy, however, still holds great promise [24,48]. A pan-HDAC inhibitor similar to SAHA, panobinostat, has demonstrated efficacy in both in vivo and in vitro models; however, has caused dose-limiting grade 4 myelosuppression in clinical trials [26,49]. Although panobinostat has demonstrated itself too toxic to be effectively administered in phase I trials, it is yet to be explored as a chemosensitizer or as intraperitoneal therapy at the time of cytoreductive surgery. Both of these applications would allow for the use of a very potent HDAC inhibitor at lower, and presumably less toxic, dosages [50]. Wilson et al. have demonstrated panobinostat to be a sensitizer of homologous recombination-proficient ovarian cancer to olaparib, a PARP inhibitor and newer treatment for HGSC [50]. As evidenced in our work, panobinostat increases the efficacy of classic chemotherapy as well. In causing spheroid shrinkage, panobinostat may decrease the size of chemoresistant clones circulating in ascites, and not only limit the spread of metastatic disease, but also make that disease more susceptible to classic chemotherapy. Even in the most persistent recurrent cell line, which demonstrates both platinum and taxane resistance, panobinostat augmented classic chemotherapy in our 3D spheroid model (Figure 12). Furthermore, if it reduces the amount of cisplatin–paclitaxel required, panobinostat can also decrease toxicity to classic chemotherapy when used sequentially.

As Modesitt et al. have suggested, the timing of epigenetic therapy with chemotherapy may be critical [44]. Sequential use of conventional chemotherapy and epigenetic drugs has been shown to decrease toxicity to normal cells while maintaining cytotoxicity in ovarian cancer cells in vitro [29]. This method has not been utilized in clinical trials involving epigenetic therapy. Other modalities, such as intraperitoneal therapy, may present an opportunity to maximize the effectiveness of epigenetic therapy while minimizing its toxicity. As Tanenbaum et al. have demonstrated in a spheroid model, intraperitoneal doses of chemotherapy may be more effective at shrinking spheroids intra-abdominally [17]. Indeed, panobinostat may be more effective upfront, as intraperitoneal therapy prior to initiating classic chemotherapy. This is the premise behind the OVATION 2 trial which is currently recruiting patients (NCT033938840). In this trial, Il-12 is being given intraperitoneally prior to neoadjuvant chemotherapy in order to increase sensitivity and act synergistically with classic chemotherapy.

Our study does have some limitations. We were quantifying growing bodies of cells over time; therefore, we could not directly use the same unit (for example, cell counts) in all of our observations. Thus, we relied on several different methods to both measure and observe the trends in our results. We directly observed the spheroids with our apoptosis–necrosis assay, again, because we were observing spheroid growth over time. However, spheroid slices, if technically feasible, would be a better way of quantifying the data. Additionally, we utilized an MTT assay to observe chemoresistance patterns in our cells. This could have been better quantified with growth curves and/or colony assays.

Future studies should expand on the spheroid model and expand the timelines in which ovarian cancer cells are exposed to treatment. In extending the kinetic of tumor testing, exploring panobinostat as an option for recurrent and persistent disease, and alternating epigenetic therapy with classic chemotherapy, our work offers new strategies for studying this deadly disease.

## 5. Conclusions

There is a crucial need to develop a 3D tissue culture system in which we have the ability to test novel therapeutic strategies in ovarian cancer treatments that may overcome this chemoresistance problem and save ovarian cancer patients. In this study, we offer a spheroid-based reliable 3D culture model that allows the side-by-side comparison of efficient and novel cancer treatments over a prolonged period of time. We anticipate that this 3D system will more closely mimic the development of cancer propagation in patients and also allow us to follow treatment outcomes for a longer time period as compared to traditional tissue culture models.

In summary, the use of paclitaxel as a single agent caused the exponential growth of recurrent ovarian cancer cell lines in our 3D culture over an extended kinetic. These changes were consistently reversible with the epigenetic therapy panobinostat. Cells treated with panobinostat become more sensitive to classic chemotherapy. Therefore, we believe that panobinostat should be explored as a chemosensitizer in advanced ovarian cancer. It was found to be too toxic when administered simultaneously with classic chemotherapy; therefore, panobinostat should be investigated as sequentially administered with classic chemotherapy both to limit the dose but also to use the drug as a chemosensitizer. Furthermore, because it does appear to affect spheroid shrinkage, panobinostat may also have utility as an upfront intraperitoneal therapy to sterilize the abdomen of miliary disease.

## Figures and Tables

**Table 1:** Drug treatments used as well as their respective mechanisms of action and clinical relevance.

Treatment	Mechanism	Relevance
cisplatin	classic chemotherapy; platinum alkylating agent	commonly used in HGSC alone or in combination with paclitaxel
paclitaxel	classic chemotherapy;	commonly used in HGSC alone or in combination with cisplatin
	taxane antimicrotubule agent	
panobinostat	epigenetic therapy; histone deacetylase inhibitor	improves efficacy of the cisplatin–paclitaxel combination in preclinical models [27]
suberoylanilide hydroxamic acid/vorinostat	epigenetic therapy; histone deacetylase inhibitor	improves efficacy of paclitaxel combination in preclinical models [28]

**Table 2:** IC_50_ concentrations of Caov-3 and Ovcar-3.

Treatment	IC_50_ for Caov-3	IC_50_ for Ovcar-3
cisplatin	7.5 µM [29]	152 µM [30]
paclitaxel	5.4 nM [29]	0.05 µM [31]
panobinostat	30 nM [32]	30 nM [32]
suberoylanilide hydroxamic acid (SAHA)/vorinostat	44.2 µm [29]	2.1 µM [33]

## Data Availability

Data supporting reported results is publicly available can be found on figshare at https://doi.org/10.6084/m9.figshare.17026937.v1.
